# Graph Neural Networks and Their Current Applications in Bioinformatics

**DOI:** 10.3389/fgene.2021.690049

**Published:** 2021-07-29

**Authors:** Xiao-Meng Zhang, Li Liang, Lin Liu, Ming-Jing Tang

**Affiliations:** ^1^School of Information, Yunnan Normal University, Kunming, China; ^2^Key Laboratory of Educational Informatization for Nationalities Ministry of Education, Yunnan Normal University, Kunming, China; ^3^School of Life Sciences, Yunnan Normal University, Kunming, China

**Keywords:** bioinformatics, graph neural networks, deep learning, omics data, network biology

## Abstract

Graph neural networks (GNNs), as a branch of deep learning in non-Euclidean space, perform particularly well in various tasks that process graph structure data. With the rapid accumulation of biological network data, GNNs have also become an important tool in bioinformatics. In this research, a systematic survey of GNNs and their advances in bioinformatics is presented from multiple perspectives. We first introduce some commonly used GNN models and their basic principles. Then, three representative tasks are proposed based on the three levels of structural information that can be learned by GNNs: node classification, link prediction, and graph generation. Meanwhile, according to the specific applications for various omics data, we categorize and discuss the related studies in three aspects: disease prediction, drug discovery, and biomedical imaging. Based on the analysis, we provide an outlook on the shortcomings of current studies and point out their developing prospect. Although GNNs have achieved excellent results in many biological tasks at present, they still face challenges in terms of low-quality data processing, methodology, and interpretability and have a long road ahead. We believe that GNNs are potentially an excellent method that solves various biological problems in bioinformatics research.

## Introduction

**FIGURE 1:**
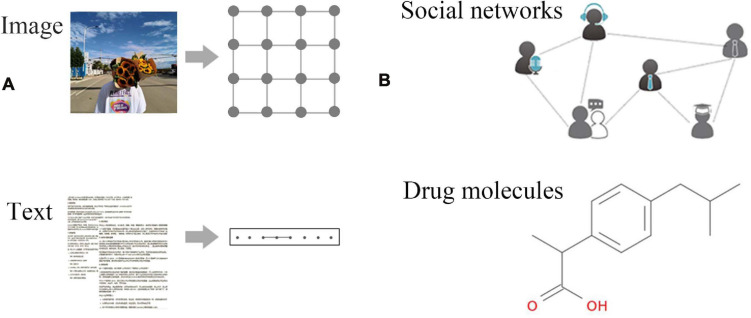
Examples of Euclidean and non-Euclidean data. **(A)** Euclidean data: regular data such as images, text, video, voice, etc. These data are characterized by excellent translation invariance, that is, the number of neighbor nodes of each node is fixed. **(B)** Non-Euclidean data: social networks, chemical molecular structures, knowledge graphs, etc.; each node has an unfixed number of neighbors.

In recent years, deep learning has met with great success in machine learning tasks such as speech recognition and image classification. Nevertheless, most of the theories of deep learning are focused on explaining regular Euclidean data (Figure 1A). With the rapid accumulation of non-Euclidean data represented by graph structure data (Figure 1B), more and more researchers begin to pay attention to the processing of graph structure data that can represent complex relationships between objects. For example, graph embedding algorithms are used to perform the mapping of graph structure data to simpler representations (Scarselli et al., 2008). However, this method may lose the topological information of the graph structure in the pre-treating stage, thereby affecting the final prediction result. Gori et al. (2005) proposed the concept of graph neural networks (GNNs) and designed a model that can directly process graph structure data based on research results in the field of neural networks. Scarselli et al. (2008) elaborated on this model, which showed that GNNs could deliver significantly better results than traditional methods due to using the topological information of graphs in an iterative process. Subsequently, new models and application research on GNNs have been proposed. With the increasing interest in graph structure data mining, the research direction and application fields of GNNs have been greatly expanded.

In general, GNNs are actually a connectionist model that captures the dependence of graphs through message passing between nodes, which take into account the scale, heterogeneity, and deep topological information of input data simultaneously. At present, GNNs show reliable performance in mining deep-level topological information, extracting the key features of data, and realizing the rapid processing of massive data, such as predicting the properties of chemical molecules (Duvenaud et al., 2015), extracting text relationship (Peng et al., 2017), reasoning the structure of graphics and images (Wang et al., 2018), link prediction and node clustering of social networks (Zhang and Chen, 2018), network completion of missing information (Bojchevski and Günnemann, 2017), drug interaction prediction (Zitnik et al., 2018), etc.

In the era of biomedicine “big data,” the aggregation and growth of large amounts of multiform data created enormous challenges to bioinformatics studies. In response to the characteristics and demands of these data, many algorithms in the field of machine learning, especially in deep learning, have been widely used in bioinformatics and propelling the development of bioinformatics. In many cases, biological data is constructed as a biological network in non-Euclidean domains, such as the molecular structure of proteins and RNAs, genetic disease association networks, and protein interaction networks. These biological networks have a great contribution to bioinformatics studies, especially for revealing the complex mechanisms of diseases. Network-based disease prediction methods had been proposed in 2011 (Barabasi et al., 2011), which was based on the assumption that “if a few disease components are identified, other disease-related components are likely to be found in their network-based vicinity.” Goh et al. (2007) pointed out that the number of interactions between proteins in the same disease pathway was 10 times higher than in random experiments. Navlakha and Kingsford (2010) proved that the network topology method was effective in predicting the association of diseases and even the interaction of biomolecules. Compared with other models in deep learning, the natural advantage of GNNs in capturing hidden information in biological networks brings new opportunities to design computational models in the biology field. Besides this, GNNs are not only suitable for non-Euclidean data but also able to extract potential graph structures from data without apparent graph structures like images and make inferences and judgments based on this structure. Therefore, GNNs have been widely adopted in the field of medical imaging.

**FIGURE 2:**
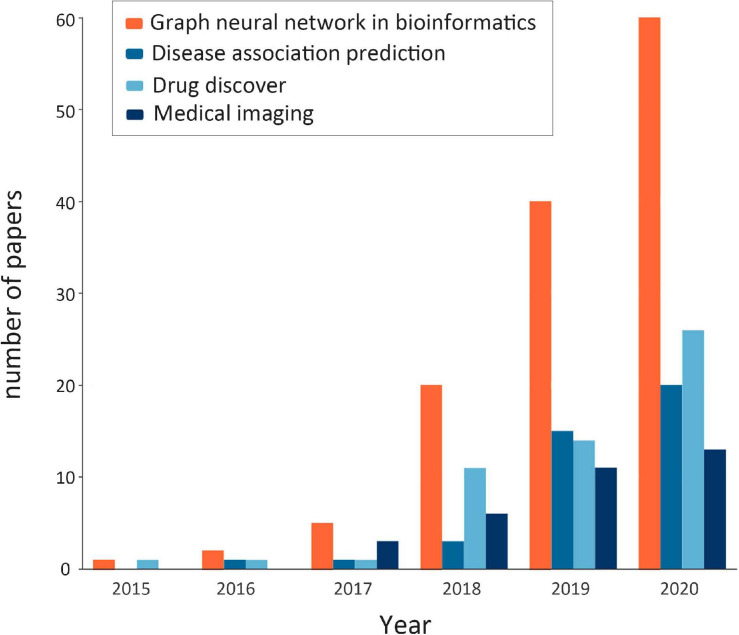
Statistics of published graph neural network (GNN) articles in bioinformatics from 2015 to 2020. The orange bar shows the total number of GNN papers in bioinformatics that year (note: the number of papers in 2020 is counted until October). The remaining colors, in turn, represent the number of papers related to GNNs in bioinformatics in terms of disease association prediction, drug research, and medical image processing, which are the components of the orange bar.

Through extensive literature investigation, we find that the application of GNNs in bioinformatics has been rapidly developed in recent years, and the number of research papers in this field has shown a rapid growth. Figure 2 shows the statistics of published GNN articles in bioinformatics from 2015 to 2020.

Although previous studies have surveyed deep learning applications in bioinformatics (Wood and Hirst, 2004; Min et al., 2016; Sun et al., 2019) recently published a review paper of GCN in bioinformatics, these studies are confined to a narrow field such as drug discovery in reference. To the best of our knowledge, this is the first effort to review the application and development of GNNs for bioinformatics. The rest of this paper is carried out from the following aspects: (1) several standard GNN models are introduced for a better understanding on how GNNs extract potential information from biological data; (2) three levels of GNN applications (node level, edge level, and graph level) are illustrated in specific biological tasks. Meanwhile, existing applications of GNNs for bioinformatics are classified based on various biological problems and data forms, and the role of GNNs in these studies is discussed; (3) according to the discussions on existing studies, we summarize the limitations of this field, including imbalances of biological data, and the methodological and interpretability challenges of GNNs. Finally, future research directions on various applications are proposed.

## Model Principle and Development

There have been various GNN models for processing graph structure data. In this section, we present the original GNN and its variant models, including graph convolutional network (GCN), graph attention network (GAT), and graph autoencoders.

### Graph Neural Network

Gori et al. (2005) proposed a novel neural network model capable of processing graph structure data–graph neural network in 2005. As a pioneering work of deep learning methods in non-Euclidean spaces, the goal of GNN is to learn how to generate an accurate state embedding vector *h_i_*, that is, the state of the node is constantly updated with the information dissemination mechanism on the graph; each update depends on the state information of the neighboring nodes at the previous time.

The related concepts are introduced as follows: let the input graph be *G* = (*V*,*E*,**X**_*V*_,**X**_*E*_), *V* = {*v*_1_,*v*_2_,…,*v*_*n*_} represents the set of nodes, and *E* = {(*i*,*j*)|when*v*_*i*_*isadjacenttov*_*j*_} is the set of edges. **x**_*i*_ denotes the feature vector of node *v_i_*, and **X**_*V*_ = {**x**_1_,**x**_2_,…,**x**_*n*_} is the set of feature vectors of all nodes. **x**_(*i*,*j*)_ denotes the feature vector of edge (*i*,*j*), and **X**_*E*_ = {**x**_(*i*,*j*)_|(*i*,*j*) ∈ *E*} is the set of feature vectors of all edges.

**FIGURE 3:**
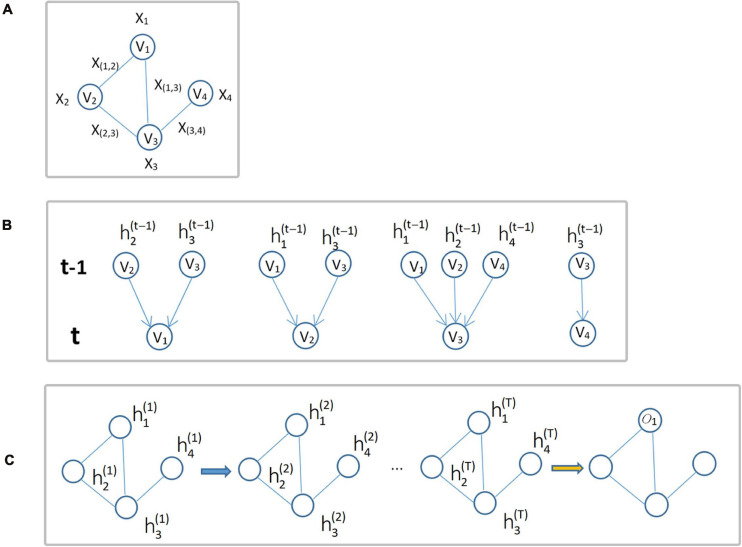
Update of node status. **(A)** Input graph structure data G. **(B)** Diagram of each node iteration from time *t*-1 to time *t*. **(C)** The overall iteration process, where *o_i_* is the output of the *i*-th node iteration.

The input graph G is converted into a dynamic graph *G*^*t*^ = (*V*,*E*,*X*_*V*_,*X*_*E*_,*H*^*t*^) in the graph neural network model, where *t* = 1, 2,…,*T* represents time and $H^{t}=\left(h_{1}^{\left(t\right)},h_{2}^{\left(t\right)},\ldots ,h_{n}^{\left(t\right)}\right)$, $h_{i}^{\left(t\right)}$represents the state vector of node *v_i_* at time *t*, which depends on the graph *G*^*t*−1^ at time *t-1*. The equation of $h_{i}^{\left(t\right)}$ is as follows:

(1) $$h_{i}^{\left(t\right)}=f_{w}\,\left(x_{i},x_{c\,o\,\left(i\right)},h_{n\,e\,\left(i\right)}^{t-1},x_{n\,e\,\left(i\right)}\right)$$

where *f*_*w*_(⋅) denotes the local transformation function with parameter *w*,*x*_*ne(i)*_ is the set of feature vectors of all nodes adjacent to node *v_i_*, *x*_*co(i)*_ is the set of feature vectors of all edges connected to node *v_i_*, and $h_{n\,e\,\left(i\right)}^{\left(t\right)}$is the set of state vectors of all nodes adjacent to node *v_i_* at time *t*. GNN updates the node status in an iterative manner, and this process is shown in Figure 3.

The long-term dependency problem of the original GNN (it is difficult for the node features to affect the state after multiple updates) makes it laborious to learn the deep structure. Based on GNN, some variant models appear successively.

### Graph Convolutional Networks

The existing GCN models can be divided into two categories: spectral-based and spatial-based GCN. Spectral-based GCN is defined from the perspective of graph signal processing, which exploits the principle of Laplacian and Fourier transform to map the irregular structure of a graph to a regular Euclidean space for convolution operation. Spatial-based GCN directly utilizes the information dissemination mechanism on the graph to define the convolution operation, and its propagation method is similar to the original GNN. These two models will be discussed next.

#### Spectral-Based GCN

Spectral-based GCN uses the graph Laplace matrix as an important tool to extend the Fourier transform to the graph structure. Let A be the adjacency matrix with weighted undirected graph G, and the element **A**(*i*,*j*) in the *i-*th row and *j* column of the matrix is the weight of the edge (*i*,*j*). Degree matrix **D** is defined as follows:

(2) $$\text{D}\,\left(i,j\right)=\sum_{j=1}^{n}\text{A}\,\left(i,j\right)$$

The symmetric normalized Laplace matrix of graph **G** is defined as follows:

(3) $$\text{L}=\text{I}-{\text{D}}^{-\frac{1}{2}}\,{\text{AD}}^{-\frac{1}{2}}$$

As a real symmetry positive semidefinite matrix, ***L*** can be decomposed into:

(4) $$\text{L}=\text{U}\,\Lambda \,{\text{U}}^{T}$$

where **U** = (**u**_0_,**u**_1_,⋯,**u**_*n*−1_) is the eigenvector matrix, and $\Lambda =\left[\begin{array}{ccc} {\text{λ}}_{\text{1}} & \ldots & 0\\ \ldots & \ldots & \ldots \\ 0 & \ldots & {\text{λ}}_{n} \end{array}\right]$ is the diagonal matrix of eigenvalues. The normalized Laplacian matrix ***L*** and its eigenvector *u* form an orthogonal space as the Fourier transform ecosystem on the graph. The graph signal represents the feature vector of all nodes in the graph, expressed by **x** = (**x**_0_,**x**_1_,⋯,**x**_*n*−1_) ∈ ***R***^*n*^. The Fourier transform of the graph signal *x* is given below.

(5) $$\hat{\text{x}}={\text{U}}^{T}\,\text{x}$$

Calculate the convolution between the two signals as:

(6) $$\text{x}*\text{g}=\text{U}\,\left(\left({\text{U}}^{T}\,\text{x}\right)\,⊙\left({\text{U}}^{T}\,\text{g}\right)\right)$$

If **g**_*θ*_ = *diag*(***U***^*T*^**g**) is used as a filter for the graph signal *x*, we can define the graph convolution as follows:

(7) $$\text{x}*{\text{g}}_{\theta }={\text{Ug}}_{\theta }\,\left(\Lambda \right)\,{\text{U}}^{T}\,\text{x}$$

This is the first generation of a spectral-based GCN model proposed by Bruna et al. (2013), which contains multiple convolutional layers. Spectral-based GCN maps the graph structure to Euclidean space through the Laplacian matrix of the graph to realize the spectral convolution of the graph. Nevertheless, due to matrix–vector multiplication, the computational complexity of the model is relatively high, which is *O*(*n*^2^). To solve this problem, Defferrard et al. (2016) proposed a model ChebNets that uses a ***K***-degree polynomial filter in the convolutional layer. The κ-th polynomial filter of the spectrum in the model is expressed as shown below.

(8) $${\text{g}}_{\theta }=\sum_{k=0}^{K}\theta_{k}\,\lambda_{l}^{k}$$

The *K*-th-order polynomial filter of the spectrum is expressed in the node domain as aggregating *K*-th-order neighborhoods to maintain spatial locality, and the number of filter parameters is also controlled to *O*(*K*) = *O*(1). In order to further reduce the computational complexity, the model uses Chebyshev polynomial **T**_*k*_(*x*) = 2**T**_*k*−1_(*x*)−**T**_*k*−2_(*x*) for recursive calculation, where **T**_0_(*x*) = 1 and **T**_1_(*x*) = *x*. Therefore, the convolution of the graph signal *x* and the filter is defined as shown below.

(9) $$\text{x}*{\text{g}}_{\theta }=\text{U}\,\left(\sum_{k=0}^{K}\theta_{k}\,{\text{T}}_{k}\,\left(\tilde{\text{L}}\right)\right)\,{\text{U}}^{T}\,\text{x}$$

As a simplification of the above-mentioned ChebNets, the graph convolutional network model proposed by Kipf and Welling truncates the Chebyshev polynomial to one time (Kipf and Welling, 2016a). For numerical stability, the adjacency matrix ***A*** is adjusted to obtain $\tilde{\text{A}}$, which results in a simplified combined convolutional layer.

(10) $$\text{H}=\text{X}*{\text{g}}_{\theta }=f\,\left({\tilde{\text{D}}}^{-\frac{1}{2}}\,\tilde{\text{A}}\,{\tilde{\text{D}}}^{-\frac{1}{2}}\,\text{X}\,\Theta \right)$$

where $\tilde{\text{A}}=\text{I}+\text{A}$, and ${\tilde{\text{D}}}_{i\,j}=\sum j\,{\tilde{\text{A}}}_{i\,j}$, *f*(⋅)is the activation function; Θ is the filter parameter matrix.

Although the above-mentioned methods based on frequency domain perform well in feature extraction, their limitations are also obvious. First of all, due to the problem of data volume, the method based on the Laplacian matrix of graphs is hard to calculate on large graphs. Second, the trained GCN can only be applied to a fixed graph structure rather than to arbitrary-structure graphs.

#### Spatial-Based GCN

The above-mentioned methods are based on the convolution theorem and define the graph convolution in the spectral domain, while the spatial method starts from the node domain and aggregates each central node and its neighboring nodes along the edge. Diffusion convolutional neural network (DCNN) (Atwood and Towsley, 2015) proposes that convolution is a process of diffusion between nodes and uses the k-hop transition probability obtained after random walking to define the weight between nodes. The structure of layer *m* is as follows:

(11) $${\text{H}}^{\left(m+1\right)}=f\,\left({\text{WP}}^{k}\,{\text{H}}^{m}\right)$$

where ***P***^*k*^ denotes the k-hop reachability probability between two nodes in a random walk, and **W** is a learnable model parameter. DCNN describes the high-order information between nodes, but it is hard to extend to a large graph because the computational complexity of the model is *O*(*n*^2^*K*).

GraphSage (Hamilton et al., 2017) randomly samples the neighboring nodes so that the neighboring nodes of each node are less than the given number of samples so as to adapt to the application on large-scale networks. The graph convolution operation is as follows:

(12) $${\text{h}}_{v}^{\left(k\right)}=\sigma \left({\text{W}}^{k}\cdot f_{k}\left({\text{h}}_{v}^{\left(k-1\right)},\left\{{\text{h}}_{u}^{\left(k-1\right)},\forall u\in S_{N_{\left(v\right)}}\right\}\right)\right)$$

where *f*_*k*_(⋅) is the aggregate function, and *S*_*N_(v)_*_ is the random sampling result of neighbors of node *v*. GraphSage gives a variety of forms of aggregation functions, which are mean aggregator, LSTM aggregator, and pooling aggregator.

There are also some studies aimed at defining the general framework of GCNs. Among them, mixture model networks (MoNet) (Monti et al., 2017) focus on the lack of translation invariance on the graph and map the local structure of each node to a vector of the same size by defining a mapping function. Finally, learn the shared convolution kernel on the result of the mapping. The message passing neural network (MPNN) (Gilmer et al., 2017) is based on information dissemination and aggregation between nodes and proposes a framework by defining a general form of the aggregation function.

Mixture model networks defines a coordinate system on the graph and expresses the relationship between nodes as a low-dimensional vector in the new coordinate system. At the same time, a weight function is defined on all adjacent nodes centered on a node, and a vector representation of the same size is obtained for each node.

(13) $${\text{D}}_{j}\,\left(x\right)\,f=\sum_{y\in N\,\left(x\right)}w_{j}\,\left(\text{u}\,\left(x,y\right)\right)\,f\,\left(y\right),j=1,\cdots ,J$$

where *N*(*x*) represents the set of adjacent nodes of *x*, *f*(*y*) represents the value of node *y* on the signal *f*, **u**(*x*,*y*) refers to the low-dimensional vector representation of the node relationship in the coordinate system **u**, *w_j_* represents the j-th weight function, and *J* represents the weight function number. This operation makes each node get a *J*-dimensional representation, and the shared convolution kernel is defined on this.

Differently from MoNet, MPNNs point out that the core of graph convolution is to define the aggregation function between nodes using the aggregation function to get the local structure expression of each node and its neighboring nodes and then applying the update function to itself and the local structure expression to get the new expression of the current node. The convolution operation is as follows:

(14) $${\text{h}}_{v}^{\left(k\right)}=U_{k}\,\left({\text{h}}_{v}^{\left(k-1\right)},\sum_{u\in N\,\left(v\right)}M_{k}\,\left({\text{h}}_{v}^{\left(k-1\right)},{\text{h}}_{u}^{\left(k-1\right)},{\text{x}}_{v\,u}^{e}\right)\right)$$

where *U_k_* and *M_k_* are update function and aggregate function, respectively. The aggregate function learned under the spatial framework can be adapted to the task and the specific graph structure and has greater flexibility.

### Graph Attention Networks

In order to solve the shortcomings of GCN and its similar structure, GAT (Veličković et al., 2017) introduces the attention mechanism into the propagation step of the graph to learn the weight between two connected nodes. In the GAT model, input the set **x** = {**x**_1_,**x**_2_,…,**x**_*n*_} of the node feature to an attention layer; a new learned set **h** = {**h**_1_,**h**_2_,…,**h**_*n*_} of node feature will be output. The attention coefficient of edge (*i*,*j*) is represented by α_*ij*_, and the equation is as follows:

(15) $$\alpha_{i\,j}=\frac{exp\left(\text{LeakyRELU}\left(a^{T}\left[{\text{Wx}}_{i}||{\text{Wx}}_{j}\right]\right)\right)}{\sum_{k\in N_{i}}exp\left(\text{LeakyRELU}\left(a^{T}\left[{\text{Wx}}_{i}||{\text{Wx}}_{k}\right]\right)\right)}$$

where *N*_*i*_is a set composed of adjacent nodes of node *v_i_*, *a* represents the learnable weight vector, and **W** is a shared linear transformation weight matrix. The output features of each node are calculated by the following equation:

(16) $${\text{h}}_{i}=\sigma \,\left(\sum_{j\in N_{i}}\alpha_{i\,j}\,{\text{Wx}}_{j}\right)$$

Multi-head attention expands the attention layer into *K* independent attention mechanisms to make the learning process of self-attention more stable, and the final expression is given as shown below.

(17) $${\text{h}}_{i}=\sigma \,\left(\frac{1}{K}\,\sum_{k=1}^{K}\sum_{j\in N_{i}}\alpha_{i\,j}^{k}\,{\text{W}}^{k}\,{\text{x}}_{j}\right)$$

Parallel computing operations give GAT a higher efficiency, and the applicability of GAT on completely unknown graphs makes up for the limitation of spectral GCN.

### Graph Autoencoder Networks

The wide application of autoencoder (AE) and its variants in the field of unsupervised learning has led to an increasing number of AE-based graph generation models. Sparse autoencoder (SAE) (Tian et al., 2014) is the source of AE-based graph neural network. It uses the following ${ℒ}_{2}$ reconstruction loss:

(18) $$\underset{\theta }{\text{min}}{ℒ}_{2}=\sum_{i=1}^{N}{||\text{P}\,\left(i,:\right)-\hat{\text{P}}\,\left(i,:\right)||}_{2},$$

(19) $$\hat{\text{P}}\,\left(i,:\right)=\text{G}\,\left({\text{h}}_{i}\right),{\text{h}}_{i}=\text{F}\,\left(\text{P}\,\left(i,:\right)\right),$$

where ***P*** is the transition matrix, and $\hat{\text{P}}$ is the reconstruction matrix; **h**_*i*_ ∈ ***R***^*d*^ represents the low-dimensional representation of node *v_i_*, and ***F*** and ***G*** are encoder and decoder, respectively. *d* is the dimension of hidden variables, and *𝒩* is the number of nodes and *d* ≪*N*. On the basis of SAE, Wang et al. (2016) proposed a structural deep network embedding (SDNE) model, which modified the reconstruction loss function as shown below.

(20) $$\underset{\theta }{\text{min}}{ℒ}_{2}=\sum_{i=1}^{N}{||\left(\text{A}\,\left(i,:\right)-\text{G}\,\left({\text{h}}_{i}\right)\right)\,⊙b_{i}||}_{2}$$

when **A**(*i*,*j*) = 0,*b*_*ij*_ = 1; otherwise, *b*_*ij*_ = *β* > 1, and β is a hyperparameter. The supervised learning method is used to learn the first-order approximation. The loss function is as follows:

(21) $${ℒ}_{1}=\sum_{i,j=1}^{N}\left(\text{A}\,\left(i,j\right)\,{||{\text{h}}_{i}-{\text{h}}_{j}||}_{2}^{2}\right)$$

Finally, the loss function of SDNE is obtained:

(22) $$ℒ={ℒ}_{2}+\alpha \,{ℒ}_{1}+{ℒ}_{\text{reg}}$$

The variational autoencoder (VAE) (Kingma and Welling, 2013) is suitable for learning graph node representation without supervision information. Kipf and Welling (2016b) proposed a variational graph autoencoder (VGAE), which was the first time that VAE was extended to graphs. The generation model of VGAE is as follows:

(23) $$p\,\left(\text{A}|\text{H}\right)=\prod_{i=1}^{N}\prod_{j=1}^{N}p\,\left(\text{A}\,\left(i,j\right)|{\text{h}}_{i},{\text{h}}_{j}\right)$$

(24) $$p\left(\text{A}\left(i,j\right)=1|{\text{h}}_{i},{\text{h}}_{j}\right)=\text{sigmoid}\left({\text{h}}_{i}^{T},{\text{h}}_{j}\right)$$

Variational graph autoencoder learns parameters by minimizing the lower bound of variation ***L***.

(25) $$\text{L}=E_{q\,\left(H|F^{V},A\right)}\left[\text{log}p\left(\text{A}|\text{H}\right)\right]-KL\text{[}q\left(\text{H}|F^{V},A\right)||p\left(H\right)]$$

Among them, *KL*(⋅) represents the Kullback–Leibler divergence function, which is used to measure the distance between two distributions. The time complexity of the model is *O*(*N*^2^).

## Application Principle of GNNs in Bioinformatics

### Modeling Methods

**FIGURE 4:**
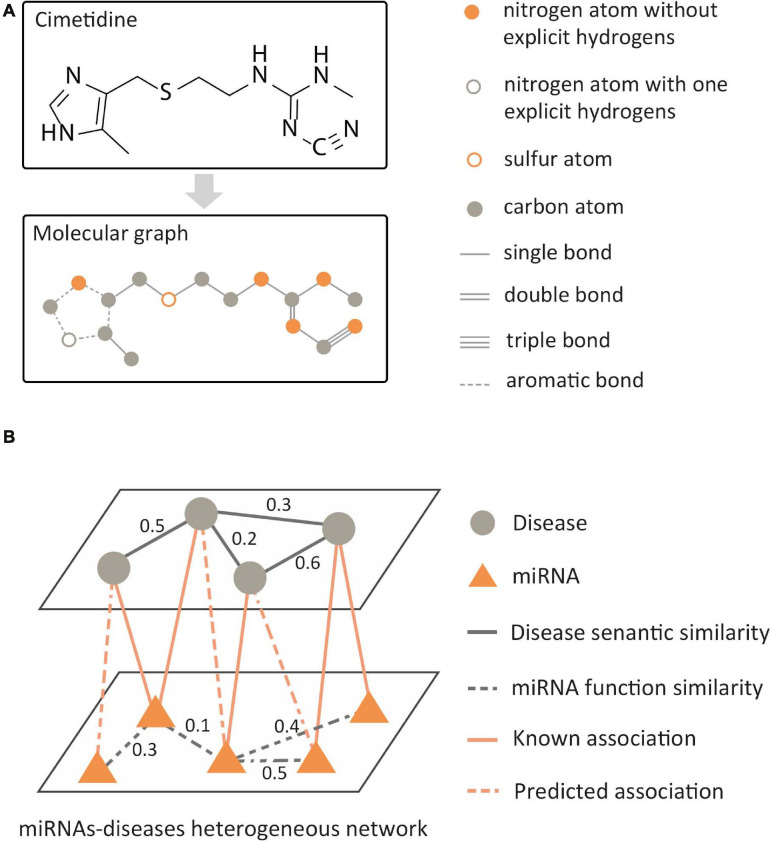
Two modeling methods of biological data with graph structure. **(A)** Molecular structure-based modeling. **(B)** Biological network-based modeling.

In the data analysis of bioinformatics, the biological data with graph structure can be modeled in two ways: molecular structure-based modeling and biological network-based modeling. For molecular structured-based modeling, atoms or valid chemical substructures (Jin et al., 2018) are used as nodes, bonds are used as edges, and then the molecular graph is constructed as shown in Figure 4A. Molecular graphs have a wide range of applications in predicting the properties of molecules and *de novo* molecular design. For biological network-based modeling, various entities are used as nodes, such as gene, disease, RNA, etc. The edges between nodes mean that there is a known association between pairs of entities, such as miRNA–disease interaction. Then, a relational network is generated, as shown in Figure 4B. GNNs are known to have a perfect performance in extracting potential information from graph structures, so they can process omics data in the biological field, including genomics, proteinomics, RNomics, and radiomics. Combined with the above-mentioned two modeling methods, applying GNNs in these omics data can be employed for a variety of tasks, such as molecular property prediction, *de novo* molecular design, link prediction, node classification in biological networks, etc.

### The Tasks of GNNs in Bioinformatics

**FIGURE 5:**
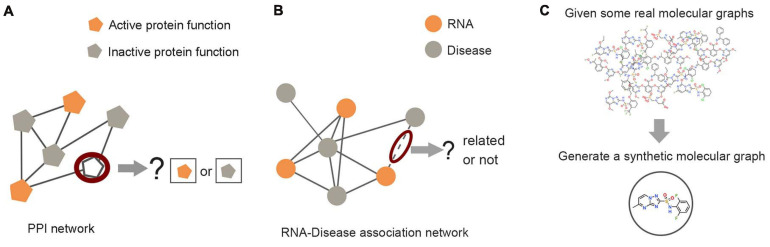
Examples of graph analysis tasks in three levels. **(A)** Node level: the prediction of unlabeled proteins through labeled proteins in the protein–protein interaction network. **(B)** Edge level: predicting the unknown link between RNA nodes and disease nodes through the RNA–disease association network. **(C)** Graph level: the generation of synthetic molecules through actual molecular graph learning.

Based on the modeling methods cited above, the structural information learned by GNNs provided a basis for different levels of graph analysis tasks: node level, edge level, and graph level (shown in Figure 5).

#### Node Level

Node classification is the typical task at the node level (Figure 5A), which can be performed by way of supervised learning, unsupervised learning, and semi-supervised learning. As the most commonly used method of node classification, semi-supervised learning combines the characteristics of supervised learning and unsupervised learning. Compared with supervised learning and unsupervised learning, semi-supervised learning on the graph extracts high-level node representations through information dissemination, which does not need to label all nodes and make good use of some known associated information. This setting is powerful for the task of inferring the association between entities in the biological network. For example, Ioannidis et al. (2019) constructed multiple protein–protein interaction (PPI) networks based on protein connectivity for different types of cells and proposed a graph residual neural network (GRNN) architecture for semi-supervised learning over multi-relational graphs. The influence of different relations was measured by learnable parameters. For the protein function prediction in generic cell, brain cell, and circulation cell data sets, GRNN had a macro F1 score of 0.86, 0.77, and 0.80, which was far better than the baseline model. In allusion to population disease prediction, Parisot et al. (2017) modeled population information as a graph, medical imaging data as the feature of the subject node, and phenotype data as the weight of the edge. GCN was utilized to simultaneously model individual features and associations between subjects from potentially large populations. In the setting of semi-supervised learning, conditioning the GCN on the adjacency matrix provides the representation learning for all nodes. Compared with the standard linear classifier, their work improved the quality of prediction.

#### Edge Level

As the main task of the edge level, link prediction is defined as, given some graphs, an edge prediction model is trained based on the features of nodes or edges for predicting the connectivity probability between node pairs in these graphs or newly given graphs, as indicated in Figure 5B. The link prediction task has captured the attention of different research fields due to its broad applicability. Predicting the interaction between biological entities from complex biological networks also plays an important role in the research of bioinformatics and has become increasingly important and more challenging. The GNN models are also effective for solving the link prediction tasks. Zhang and Chen (2018) proposed the SEAL (learning from subgraphs, embeddings, and attributes for link prediction) model based on information dissemination, which used GNN to replace the fully connected neural network in the traditional Weisfeiler–Lehman neural machine method and learned general graph structure features from local subgraphs. Its performance on public biological network data sets such as yeast, *Caenorhabditis elegans*, and *Escherichia coli* was superior to the traditional graph embedding models. In addition, GCN has been utilized to predict various interactions in biological networks. For example, PPI networks with a small amount of label information that were encoded to predict the relationship between drugs and diseases (Bajaj et al., 2017), disease similarity networks, and microRNA (miRNA) similarity networks were built to indicate the association between miRNA and disease by VGAE (Ding et al., 2020).

#### Graph Level

**FIGURE 6:**
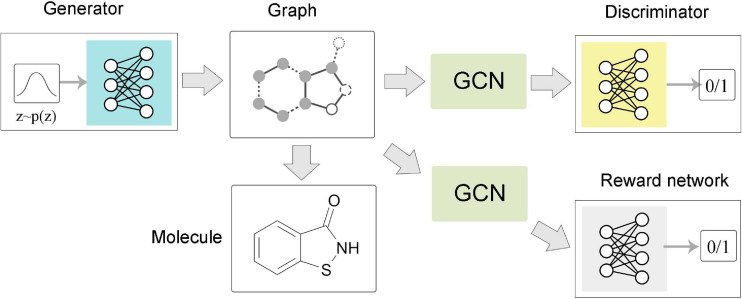
Flow chart of MolGAN. The generator extracted a sample from the prior distribution and generated a molecular graph corresponding to a specific compound. The graph neural network-based discriminator and reward network directly operate on the representation of the graph structure. The discriminator learns to distinguish whether the molecular graph comes from the real data set or the generator. The reward network assigned rewards to each molecular graph based on samples and optimized the generation process through reinforcement learning methods.

The task of graph level is mainly related to graph generation (Figure 5C). Learning to generate graph structure data by training on a set of representative data is the core of graph generation tasks. For discovering new chemical structures, a graph generative model based on GNNs was first proposed with the motivation of molecular graphs generation. Simonovsky and Komodakis (2018) combined GNN and VAE to propose GraphVAE, which was used for small-scale molecular graph generation. Experiments on the QM9 database and ZINC database proved that GraphVAE has a higher accuracy than the previous methods. Jin et al. (2018) proposed a junction tree variational autoencoder (JT-VAE), which allowed the model to gradually expand the molecule while maintaining the chemical validity of each step. The experimental results on the ZINC database had shown that JT-VAE could generate better results than the traditional model and GraphVAE. MolGAN (De Cao and Kipf, 2018) is a generative model for small graphs, which is able to generate discrete graph structures and promote the generation of molecules with specific chemical properties through reinforcement learning methods (Figure 6). The MolGAN model produced nearly 100% effective compounds in experiments on the QM9 chemical database. In practice, the abilities of graph generative models for protein structure prediction and chemical molecular map generation play an important role in related applications, such as drug design and protein structure design.

In general, as a novel type of graph embedding method, GNNs can perfectly integrate the features of nodes and the structural information between nodes in various specific applications. To better illustrate the application status and mechanisms of GNNs in bioinformatics, more comprehensive existing research on these three levels are summarized for specific biological problems, which are discussed in the next section.

## Typical Application of GNNs in Bioinformatics

Based on various biological tasks, the existing application of GNNs in bioinformatics can be categorized into three typical topics: disease association prediction, drug development and discovery, and medical imaging. Note that these applications are also based on the three levels of graph analysis tasks in the previous section. In this section, the way and the development of GNNs handle representative problems are described in more detail.

### Disease Association Prediction

Discovering the associated factors with various diseases is an important task in bioinformatics. At present, the existing methods of disease association prediction mainly include matrix decomposition (Koren et al., 2009; Wang et al., 2017) network propagation (Lee et al., 2011; Guan et al., 2012; Li and Li, 2012; Sun et al., 2014; Zhou et al., 2015), and machine learning (Luo et al., 2016; Zhou and Skolnick, 2016; Frasca, 2017; Xuan et al., 2019b; Jiang and Zhu, 2020). Essentially, some machine learning methods are also based on similarity measures and matrix decomposition. Nevertheless, matrix factorization methods map the features of entities to a latent space but ignore the representation of topological relationships between entities. In other methods, the shallow models ignore the rich structural information in disease-related networks, which ultimately affects the quality of entity feature representation. Recently, GNNs have been used to capture the nonlinear relationship between diseases and other entities in biological networks. More and more methods have introduced convolution operations into heterogeneous networks for extracting features of local sub-graphs. All of the studies discussed in this section have directly or indirectly contributed to the development of deep learning methods in the field of disease prediction. Different biological networks were constructed in these studies, which were based on RNA–disease associations, disease–gene associations, and other association information.

#### RNA–Disease Association

A large amount of evidence has shown that microRNAs (miRNAs), long non-coding RNAs (lncRNAs), circular RNAs (circRNAs), and Piwi-interacting RNA are widely involved in the occurrence and development of diseases (Xuan et al., 2019a; Li J. et al., 2020; Wang L. et al., 2020; Zheng et al., 2020). Therefore, the identification of these RNA–disease associations plays a crucial role in exploring the pathogenesis of complex diseases. The RNA and disease data analysis methods based on computational models make up for the high cost and time-consuming defects of biological experimental verification methods.

**FIGURE 7:**
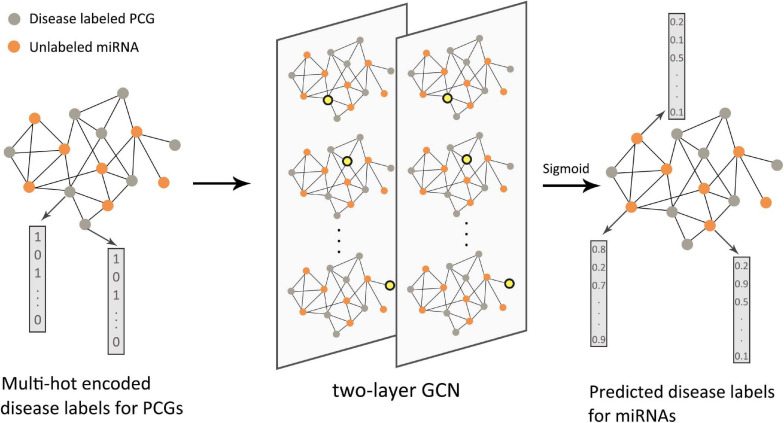
Flow chart of DimiG in RNA–disease association. The protein-coding gene (PCG)–miRNA association network was used as the input of the two-layer graph neural network. The PCGs were labeled, and the miRNAs were unlabeled. The yellow nodes in the network represented the weighted sum of neighbor embeddings. The final output could infer the probability between diseases and unlabeled miRNAs.

Starting in 2019, GNNs have been introduced into this type of research. Pan and Shen (2019) proposed a semi-supervised multi-label graph convolution model (DimiG), which did not rely on known association information between miRNAs and diseases. DimiG integrated multiple networks related to protein-coding genes and used network knowledge transfer to indirectly predict the association between miRNAs and diseases; taking DimiG as an example, Figure 7 shows how GCN uses the associated information in the network to generate features for unlabeled nodes. Li J. et al. (2020) used GCN to learn the feature representations of miRNAs and diseases from the miRNA functional similarity network and disease semantic similarity network, respectively, and utilized the neural induction matrix to generate an association matrix, combining known miRNAs and disease association information to train the model. This model can predict all miRNAs related to breast cancer without any known related miRNAs. Based on the similarity method, the association prediction between RNAs and diseases can also integrate more useful information. Li C. et al. (2019) integrated miRNA–disease, miRNA–gene, disease–gene, and PPI networks. Furthermore, based on the information extracted by GCN, the top 10 unknown interactions between miRNAs and diseases were analyzed. Using the FastGCN algorithm and the Forest by Penalizing Attributes (Forest PA) classifier, Wang L. et al. (2020) can accurately predict potential circRNA disease associations. In order to better learn the hidden representation of node features, Zhang J. et al. (2019) used GCN combined with an attention mechanism to extract domain features and conducted experimental tests on two different RNA disease networks. There have also been some studies that used the autoencoder method on the graph to reconstruct node features. Wu et al. (2020) used GCN as an encoder to learn the feature representation of lncRNAs and diseases from the bipartite graph associated with lncRNA–disease, and the score of the lncRNA–disease interaction was calculated from the inner product of the two potential factor vectors. In the research of Ding et al. (2020), VGAE was used to reduce the noise effect caused by randomly selecting negative samples. In the prediction of disease-related RNAs with limited known data, the integration of multi-view information can help us understand complex biological networks more comprehensively. Therefore, to capture a deeper interaction mode between multiple related data, the integration method of different types of data by graph deep learning model needs to be further explored.

#### Disease–Gene Association

Single-cell RNA sequencing technology provides gene expression data for a single cell. GNNs can infer the interaction between cells (Jiahua et al., 2020; Zeng et al., 2020; Wang et al., 2021) and simulate cell differentiation (Bica et al., 2020) and disease state prediction (Ravindra et al., 2020). Precise gene–disease association prediction can help researchers reveal the function of disease-causing genes and provide evidence for disease prevention. Prioritizing candidate genes for various diseases is able to accelerate the development of early treatments and solve the DGP problem to a certain extent. Rao et al. (2018) proposed a rare disease gene sequencing method, which was different from the previous association network method. They integrated the combination pairwise ontological and curated associations into a heterogeneous network and used the frequency qualifier from Orphanet to calculate the edge weights. The qualifiers included terms such as “obligation,” “very frequent,” and “frequent.” Since the learning algorithm outlined in the standard VGAE does not focus on learning the relationships between different node types, Singh and Lio (2019) proposed a constrained VGAE variant for predicting specific node associations on the disease–gene association network by improving the optimization objectives of an algorithm. Wang et al. (2019a) defined a new cluster loss function and a dropout mechanism based on the GCN and graph embedding method to improve the generalization ability.

Although a large amount of medical data has been reserved in various database, accurate prediction of cancer remains a challenge. As a group of complex diseases, cancer is caused by multiple gene defects, and there is synthetic lethality between genes. Therefore, the interaction network of genes plays an important role in cancer prediction (Iglehart and Silver, 2009). In order to analyze the underlying mechanism of cancer, Schulte-Sasse et al. (2019) initially used GCN to classify and predict cancer genes. Layer-wise relevance propagation was used to identify the gene input signals and the network topology of the learned model, which is in the neighborhood of a gene. The synthetic lethality between genes is extremely sparse. In order to solve the over-fitting problem, Cai et al. (2020) proposed a new GCN model based on fine-grained edge dropout and coarse-grained node dropout to reduce the over-fitting in sparse graphs. Chereda et al. (2021) combined the PPI network and gene expression data for patients and utilized GCN to classify the nodes in the patient’s sub-network for predicting breast cancer metastasis. In the classification of breast cancer subtypes, there are also related studies based on local GCN, which was used to combine with the PPI network and the gene expression matrix information of multiple patients (Rhee et al., 2018). The correlation generated by the GNNs for each data point not only improves the interpretability of the model but also makes it more advantageous in predicting tasks related to patient-specific disease networks.

#### Others

In addition to the studies listed above, GNNs are also introduced into some research of other related fields, for instance, the discovery of disease proteins (Eyuboglu and Freeman, 2004). The disease protein prediction problem can naturally be defined as a semi-supervised classification problem on the protein–protein interaction network. The realization of the neighborhood positioning for visualized disease pathways proved that most diseases do not have obvious neighborhood positioning. Some studies utilized graph structures to model RNA secondary molecular structures for RNA classification (Rossi et al., 2019) and RNA-binding proteins prediction (Uhl et al., 2019; Yan et al., 2020), where bases were considered as nodes in the graph and phosphodiester bonds and hydrogen bonds were two different types of edges. In other studies, miRNA, lncRNA, and other elements were used to construct heterogeneous networks for predicting the interactions between miRNA and lncRNA as well as lncRNA-targeted genes. For multi-group biomedical data classification, a weighted patient similarity network was constructed based on various omics data and cosine similarity method (Wang T. et al., 2020), and GCN performs a feature extraction on these networks so as to find the cross-omics correlation in label space for integrating multi-omics effectively.

### Drug Development and Discovery

The drug development process mainly includes drug target determination, lead compound discovery and optimization, candidate drug determination, preclinical research, and clinical research (Vohora and Singh, 2017). However, the lack of drug targets, the poor clinical transformation of animal models, disease heterogeneity, and the inherent complexity of biological systems have made drug development a long and arduous process. The purpose of modern drug development is to speed up the intermediate steps through machine learning methods so as to save development costs. Therefore, more and more researchers tend to utilize machine learning models for predicting early molecular properties, which can tremendously reduce the workload of later experiments. As the most concerning machine learning method, deep learning in the field of biomedicine showed the following limitations: first of all, most deep learning models cannot learn structural information directly from the original input data, which rely on high-quality, labeled data sets, and secondly, traditional CNN or other deep models have difficulties in directly processing unstructured data like molecular graphs, so the internal structure information of molecules is usually not fully taken into account. Therefore, GNNs, which extend deep learning methods to non-Euclidean domains, have become the latest method to deal with drug-related tasks.

#### Protein Structure and Function Prediction

Protein function research occupies an important position in life sciences, and most diseases are closely related to protein dysfunction. Anfinsen (1973) found that the denatured ribonuclease that only retained the primary structure could refold and restore biological activity, which indicated that the amino acid sequence representing the primary structure of the protein contains important information about the secondary and tertiary structure of proteins. At present, significant progress has been made in protein structure prediction. The most accurate structure prediction can fully clarify the biological mechanism of protein action on a molecular scale, and its application in drug development and other fields is of great significance to biochemical research.

High computational cost and interpretability are problems in common methods of molecular structure analysis, such as 3D CNN and 2D CNN. In recent years, some studies have shown the powerful capabilities of GNNs in learning the effective structure of proteins from simplified graphical representations. Zamora-Resendiz and Crivelli (2019) proposed a protein structure learning method that was more suitable for large data sets. Unlike the previous 3D and 2D representations, this model could apply to the natural spatial representation of molecular structures, which brought a high transferability to the application direction. Aiming at the inverse protein folding problem, Ingraham et al. (2019) proposed a protein design framework based on a similar graph attention method, which could construct a conditional generation model for a given target structure protein sequence directly, and greatly improved the design efficiency. For protein function prediction, there are two types of methods: based on protein structure (Ioannidis et al., 2019) and based on PPI networks (Gligorijevic et al., 2019). Similar to a previous work, Gligorijevic et al. (2019) modeled the protein structure as a graph to predict the protein function. Ioannidis et al. (2019) used a multi-relation diagram method based on PPI network modeling with semi-supervised learning. The structural characteristics of a protein determine the breadth and complexity of its function. However, the large number of invalid fragments contained in the protein sequence may affect the judgment of its function. Using GNNs to integrate the feature of protein relationship networks is one of the ways to solve the problem of differences in protein sequences and functions.

#### Protein–Protein Interaction Prediction

Protein–protein interaction information is able to provide theoretical assistance for drug development indirectly. Fout et al. (2017) proposed a space-based convolution operator to predict the interface between protein pairs, which was suitable for graphs with any size and structure. In the identification of protein complexes, the high rate of “false positive/negative” in the PPI network makes the detection of protein complexes arduous. Therefore, Yao et al. (2020) proposed a denoising method based on a variational graph autoencoder. They embedded the PPI network into the vector space through multi-layer GCN and deleted some interactions with credibility below the threshold so as to obtain a reliable association network. The experimental results on multiple datasets showed that the recognition accuracy of protein complexes increases by 5–200%. Liu X. et al. (2020) used an unsupervised GNN to predict the changes in protein binding properties after mutations and recognized abnormal interactions between atoms without annotations. By improving on the existing sorting algorithm, Cao and Shen (2020) and Johansson-Åkhe et al. (2020), respectively, proposed a scoring mechanism for the evaluation of protein docking models and doctored peptides. The convolution operation on graph encodes the structure and features of protein into the graph embedding representation and aggregates information along the edges of the network nodes for association scores, which solves the spatial limitations of conventional convolution methods.

#### Ligand–Protein (Drug–Target) Interaction Prediction

Drug targets are relevant to the pathological state of diseases or biomolecules, so the identification of drugs and their targets is the core problem in the development of new drugs. Drug target interaction prediction is essentially the interaction prediction problem between ligands and proteins, and many related studies have been performed. Nonetheless, there exist some problems as follows: (1) Traditional machine learning algorithms express the prediction results in a binary classification, but the real association relationship is not limited to the binary level. For some target proteins that do not exist in the test set but appear in practical applications, the prediction accuracy cannot be grasped; (2) It is more difficult to deal with the chemical space where drug molecules can be synthesized; (3) The prediction results lack biological interpretation. Although the test results of the model seem good, it is still unconvincing; and (4) The information about the ligand bound to a specific protein is always easy to obtain, but there is a lack of data on the real negative ligand–protein relationship for learning.

In response to these existing problems, Feng et al. (2018) introduced GCN into drug target identification for the first time, which learned the molecular structure information of drugs, and combined protein information as input. This study realized the prediction of the real-valued interaction strength between drugs and targets and solved the cold-target problem. There are also some studies similar to the general thinking of the above-mentioned method but differ in data processing (Gao et al., 2018; Nguyen et al., 2021). Miyazaki et al. (2020) provided a drug–target interaction prediction model that ligands were specifically targeting toward proteins without using true negative interaction information. Torng and Altman (2019) established an unsupervised graph autoencoder to learn the representation of protein pockets without relying on the target–ligand complexes, where features were extracted from the pocket graph and 2D ligand graph by GCN, respectively. Jiang et al. (2020) proposed an association prediction method that constructs a molecular graph and a protein contact graph. These graphs were based on the structural information of drug molecules and the sequence information of proteins, which were performed by a three-layer GCN for providing an accurate prediction.

Unlike the above-cited methods of representing drugs as graphs, Zhao et al. (2021) proposed a network-based prediction model that incorporated the drug–protein association network into existing methods. The feature information of each drug–protein pair learned by GCN was used as the input of a deep neural network for predicting the final label. In order to make up for the ignorance of cellular context-dependent effects in previous studies, Zhong et al. (2020) used the information in gene transcriptional profiles to indirectly predict drug-targeted binding.

Besides this, knowledge graphs are also critical in biomedicine, which can be processed by GNNs. In these studies, the result of all graph-based interaction predictions was inseparable from the quality of the knowledge graph, but the content of the knowledge graphs extracted in real life is complex and contains interference information. Therefore, Neil et al. (2018) proposed a model that could adapt to noise data and reduced the influence of noisy data on the overall prediction effect of the model by assigning low weights to unreliable edges.

#### Prediction of Molecular Properties

The prediction of molecular properties is a basic and important part of drug development. The first work to introduce graph convolution into the field of molecular properties learning (Duvenaud et al., 2015) was based on extended-connectivity circular fingerprints (ECFP), which created differentiable fingerprints to replace discrete operations in circular fingerprints and replaced hash functions with single-layer neural networks. The experimental results showed that, when the weights were large and random, this model exhibited a similar performance to ECFP, and as the weights were adjusted through training, its performance was better than ECFP. In view of the large space requirements of fingerprint-based methods and the large information noise of fingerprint encoding, Kearnes et al. (2016) proposed a molecular graph convolution method based on deep neural networks instead of molecular fingerprints. The molecular structure was represented by molecular graphs, and the distance between graphs forms the level of molecules. Although this method is not always superior in performance over molecular fingerprinting methods, it opens a new path for molecular property prediction. In addition, the emergence of multi-task deep neural networks (MT-DNNs) makes neural networks more powerful in drug discovery. Liu et al. (2019) combined GCN with MT-DNNs to further improve the prediction accuracy and realized a completely data-driven deep learning method that did not rely on domain-specific feature descriptors or fingerprints for drug property prediction. It is well known that the electrostatic calculations are useful for the prediction of the chemical reactivity of molecules and their ability to form certain types of interactions. Rathi et al. (2019) proposed a method to generate electrostatic potential surfaces close to quantum mechanics quality for ligand molecules within the time frame of interactive drug design, which provided an effective tool for medicinal chemists and modelers. In the process of drug discovery, the false positives or false negatives of bioassay conclusions caused by unstable compounds in storage make it hard to complete the stability prediction of compounds. Differently from the traditional rule-based method, Li et al. (2019b) proposed an end-to-end, attention-based GCN model to predict the stability of compounds. The model dynamically learned structural information from molecular graphs instead of pre-defined structural features, thereby reducing the risk of false alarms. The graph convolution operation can capture the local features of molecular sub-structure effects, thereby generating accurate global descriptors from the composite structure data.

#### *De novo* Molecule Design

The ultimate goal of drug design is to discover molecules with ideal chemical properties. Nonetheless, the hugeness and complexity of chemical space and the discontinuity of the spatial structure of compounds make it demanding to explore the chemical space of new molecules. By reducing the consumption of labor costs, computer-aided drug design is dedicated to accelerating the process of *de novo* molecular design. Although the generative model in machine learning can effectively generate molecules based on SMILES strings (Weininger, 1988), it cannot effectively represent the topological information of molecular structure. Based on the above-mentioned analysis, GNNs can be directly used to generate molecular graphs. Therefore, GNN is a kind of high-precision and low-cost method to determine molecular properties by analyzing the topological information of graphs.

**FIGURE 8:**
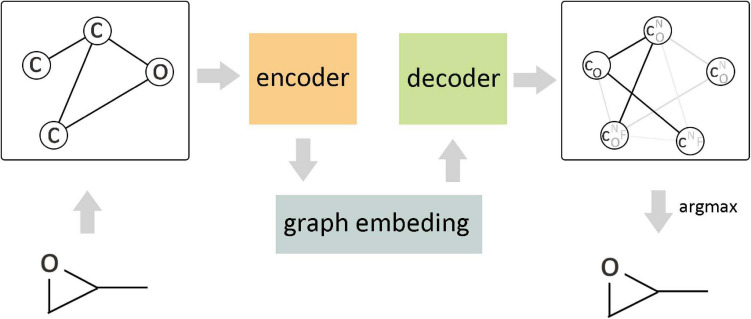
Variational graph autoencoder (VGAE) in molecular graph generation. As the encoder of VGAE, the edge condition convolution embedded the original graph into a continuous vector space, and the decoder generated a probabilistic fully connected graph according to the predefined number of nodes and updated the parameters through approximate graph matching to improve the reconstruction ability of the autoencoder.

The arbitrary connectivity and discrete structures of the graph make it laborious to generate a graph from the vectors in continuous code space, but if the maximum number of nodes in the generated graph is constrained, it is still computationally controllable. Figure 8 shows a continuous embedding method of VAE to generate small molecular graphs, which was proposed by Simonovsky and Komodakis (2018). This early generation mode avoids the difficulties that may be encountered in generating graphs; the upper bound of negative log-likelihood was minimized in the model training, but it only applies to the generation of small molecules. Another way is based on the probability description of GCN to generate the graph gradually (Li et al., 2018a) rather than directly generating the entire graph. Compared with the routine method, this method has achieved better results, but there are still challenges in the generation of macromolecular graphs. Li et al. (2018b) proposed a conditional graph generation model and explored two types of graph generation architectures. One treated graph generation as a Markov process, and the other introduced molecular-level recursive units to increase the scalability of the model. You et al. (2018) combined the prior knowledge of the example molecule dataset to generate goal-directed molecules. This method integrated and expanded three ideas of graph representation, reinforcement learning, and adversarial training, where reinforcement learning and confrontation training were combined to form a unified framework so as to reach the desired goal by continuously guiding the generation process and limiting the output space according to basic chemical rules. The experimental results show that this method achieves the most superior performance in terms of optimizing chemical properties and constrained properties under conditions similar to known molecules. Differently from the node-by-node method of generating graphs, Jin et al. (2018) proposed a method of connecting tree self-encoding, which utilized effective sub-graphs as components to generate molecular graphs in two stages. The first stage generated a connection tree structure as a sub-graph component, and then these sub-graphs were combined into a complete molecular graph in the second stage. This model avoided the generation of invalid intermediate states of molecules and improved the work efficiency. Khemchandani et al. (2020) learned the interactive binding model from the binding data through GCN and proposed an attribute prediction module, which utilized a scoring mechanism to determine the more useful molecules with the specific attribute during the generation process.

At present, the graph-based method of molecular generation has more advantages than the grammar-based generation method. Although the new compound obtained by the molecular graph method has higher scores on various evaluation indicators, it has also been drawn into question. Besides this, the method of generating molecular graphs is still limited to 2D space, and the 3D information of molecules is completely ignored, which may become the focus in the future.

#### Drug Response Prediction

The combination of genomics data and drug information for drug response prediction has promoted the development of personalized medicine. Huang et al. (2020) combined GCN with autoencoder to predict the association between miRNA and drug resistance. In this study, the association prediction was regarded as a problem of semi-supervised learning, and a graph convolution model was built by combining the known miRNA expression profile and the drug structure fingerprint information. There are some other studies that focus on the effect of drug treatment on cell growth. Liu Q. et al. (2020) predicted the therapeutic effect of drugs on cancer cells by constructing a cancer cell information sub-network and a drug structure sub-network. Considering the complexity of cancer factors, Singha et al. (2020) integrated biological network, genomics, inhibitor analysis, and disease–gene association data into a large heterogeneous graph. Multiple graph convolution blocks and attention propagation were used to aggregate network topology information, and a graphic readout framework was constructed for predicting the final result. Hwang et al. (2020) adopted a similar structure with the study of Liu et al. (Huang et al., 2020), but introduced a set of information about the dosage and duration of drug administration for predicting drug-induced liver injury.

#### Drug–Drug Interaction Prediction

When a drug is taken with another drug, the expected efficacy of drugs may be significantly changed. Therefore, research on drug–drug interaction (DDI) is essential to reduce the occurrence of adverse drug events and maximize the synergistic benefits in the treatment of diseases. Obviously, the most practical way to explore the medicinal properties of drug combinations is computer-aided DDI detection. Zitnik et al. (2018) predicted the side effects between drugs from a multimodal heterogeneous network that was composed of PPI, drug–protein targets, and drug–drug interactions, where each side effect was represented by a different edge. Ma et al. (2018) proposed a framework of a multi-view drug graph encoder based on the attention mechanism, which was used to measure the drug similarity. To make full use of the heterogeneous correlation between different views, each type of drug feature had been considered as a view, which was associated with a learnable attention weight in the similarity integration. This multi-view method could capture more similar information than the previous single view.

### Medical Imaging

Medical images play an extremely important role in clinical disease diagnosis, classification, and treatment. In the field of medical imaging, deep learning methods combined with a computer-aided diagnosis are used for the early detection and evaluation of diseases. Similar to other image-related tasks, segmentation, classification, and recognition are the main tasks of concern in medical imaging. Meanwhile, image data can be represented as a graph structure appropriate for the use of GNNs. Therefore, GNNs have an extensive application space in the field of medical imaging, such as image segmentation (Gopinath et al., 2019; Wang et al., 2019b; Tian et al., 2020a,b), abnormal detection (Wu et al., 2019) of MRI images and pathological images, classification (Shi et al., 2019; Zhou et al., 2019; Adnan et al., 2020) and visualization (Levy et al., 2020; Sureka et al., 2020) of histological images, analysis of surgical images (Zhang et al., 2018), image enhancement (Hu et al., 2020), registration (Hansen et al., 2019), retrieval (Zhai et al., 2019), brain connection (Ktena et al., 2017, 2018; Li X. et al., 2019a; Mirakhorli and Mirakhorli, 2019; Grigis et al., 2020; Li et al., 2020; Zhang and Pierre, 2020; Zhang et al., 2021) and disease prediction (Parisot et al., 2017; Kazi et al., 2018, 2019a,b,c; Anirudh and Thiagarajan, 2019; Yang et al., 2019; Stankevičiūtė et al., 2020; Zhang and Pierre, 2020; Zhang et al., 2021), etc.

#### Image Segmentation

Before GNNs were introduced, CNN-based image segmentation technology that was widely used in various studies has been maturing. Ma et al. (2018) proposed a gated graph neural network for segmenting 3D images and utilized directed graph learning to predict the movement of points on the basis of the coarse segmentation, where a second segmentation was performed to obtain a smooth image. For processing surface data (such as MRI), Tian et al. (2020a) utilized spectral convolution for realizing cortical surface parcellation. The traditional spectral embedding can only be realized in orthogonal grid space, but this method was able to directly learn the surface features of the cortex. Gopinath et al. (2019) proposed an automatic and interactive prostate contour prediction method based on GCN blocks, which could consider the multiscale feature, and utilized a contour matching loss training method to preserve the details of the prostate boundary. In most cases, the segmentation model of deep learning adopts the pixel-wise segmentation method with high computational complexity. In contrast, the GNN method only uses object contours to segment, reducing the amount of calculation.

#### Brain Connectivity Research

**FIGURE 9:**
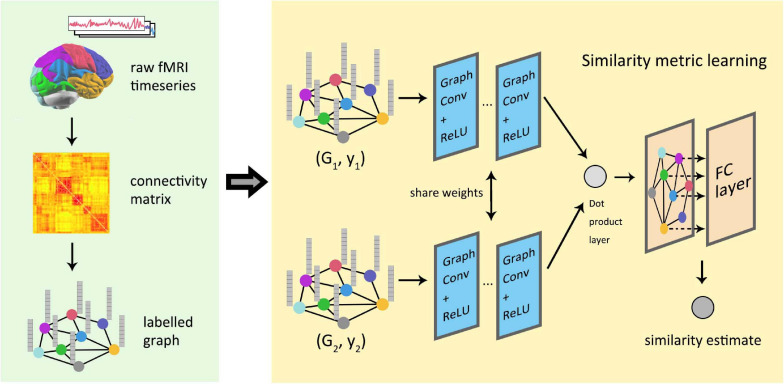
Similarity measure learning of graph neural network in functional brain graphs. The information contained in the fMRI image was integrated into the graph through the graph partition and connection matrix. Two graphs were, respectively, used as the input of the two graph convolutional networks with sharing parameters, and the output of the network was combined by the inner product layer. Finally, a fully connected layer outputs the estimated similarity between the graphs.

Global similarity measurement between graphics represents the structural or functional connections within the brain by labeled maps, which is of great significance in the study of brain connectivity. At present, most models in deep learning use regular images as the default input data, but the processing of irregular brain connection images remains a problem. The pioneering work of depth graph networks in brain connection research was done by Ktena et al. (2018), and then this work was further extended about the way of cross-validation (Mirakhorli and Mirakhorli, 2019). Figure 9 shows the process for the conversion and analysis of functional magnetic resonance imaging (fMRI) in the study of Ktena et al. which has been used in some later research of GNNs for brain connection. Zhai et al. (2019) constructed a generative model using graph convolution and VAE to predict the abnormal parts of input graphs. In order to lift the restrictions of fixed graphic structure for a model, Zhang and Pierre (2020) proposed a spatial GCN-based learning model, which could perform the classification of different brain connections and help predict the association between brain connection sub-networks and disease. Parisot et al. (2017) proposed an effective method to annotate human brain activity and then conducted a study on the transferability of brain decoding (Stankevičiūtė et al., 2020). Yang et al. (2019) predicted the state of consciousness of the brain by constructing a dynamic functional connectivity matrix that describes the state of the brain, which proved the effectiveness of graph convolution methods in predicting cortical signals.

#### Multimodal Fusion

All the studies discussed above are based on the analysis of a single image. Nevertheless, using only single-modal imaging data in disease prediction may lead to a lack of precision in the results. Yang et al. (2019) used the weighted edge-weighted graph attention network model to combine different modalities of medical imaging (brain structural magnetic resonance imaging and fMRI) for identifying bipolar disorder. In addition, more research tends to combine medical imaging data with non-imaging data in disease prediction. Based on multimodal fusion, the lack of some important information in monomodal data can be optimized and complemented to a certain extent. Parisot et al. (2017) introduced GCN into group-level medical applications for the first time. In this work, they proposed a population graph, which modeled people as nodes in the graph, and the feature vector of brain image was used as the feature representation of the node. Phenotypic data were combined for disease prediction in a semi-supervised way, such as gender, age, etc. In 2018, Parisot et al. extended this work with further in-depth analysis methods and modeling options (Stankevičiūtė et al., 2020). Since then, the use of population graph methods for disease prediction has become the choice of many researchers. Kazi et al. (2018) used a multi-level parallel GCN model to optimize the extraction of correlation information between nodes, which introduced an automatic learning layer for weight distribution and the attention mechanism for utilizing the features of each multimodal data (Kazi et al., 2019c). Aiming at the problem of insufficient feature extraction caused by fixed neighborhoods in GCN model, InceptionGCN (Kazi et al., 2019a) was proposed by Kazi et al., which considered the receptive field convolution kernels of different dimensions and utilized two aggregation methods to process all the features obtained by a convolution kernel. Nevertheless, the performance of this model on various datasets is quite different, so the LSTM-based attention mechanism was introduced in later work to better integrate multi-modal data (Kazi et al., 2019b). For the research of autism spectrum disorder classification problem, Anirudh and Thiagarajan (2019) proposed a more robust method, which was based on the set of weakly trained G-CNN for reducing the model sensitivity to the choice of graph construction. Arya et al. (2020) believed that population phenotypic data is not suitable for defining the relationship between edges; then, the actual similarities between the brain’s structures were used to directly extract the variables from brain MRI so as to establish the relationship between nodes. Stankevičiūtė et al. (2020) used a population graph to predict brain age but obtained unsatisfactory results. The topological characteristics between the various regions of the brain make GNNs more suitable for identifying related patterns of brain disease and effectively assisting in exploring the mechanism of the disease.

## Summary on Typical Applications

In general, the applications of GNNs in node level, edge level, and graph level are not completely independent. According to the different levels of tasks and biological problems, we give a detailed summary on the above-mentioned typical applications in Table 1.

**TABLE 1:** Summary of typical applications.

Classification	Biological problems	Publications	Task of graph neural networks		
			Node level	Edge level	Graph level
Disease association prediction	RNA–disease association prediction	Wang L. et al., 2020	√		
		Li C. et al., 2019; Zhang J. et al., 2019; Ding et al., 2020; Li J. et al., 2020; Wu et al., 2020; Zheng et al., 2020		√	
	Inter-cell interaction prediction	Wang et al., 2021		√	
	scRNA-Seq clustering	Zeng et al., 2020	√		
	Impute the dropout events in scRNA-Seq data	Jiahua et al., 2020		√	
	Simulate cell differentiation	Bica et al., 2020			√
	Disease state prediction	Ravindra et al., 2020	√		
	Disease genetic prioritization	Rao et al., 2018		√	
		Li Y. et al., 2019		√	
	Disease–gene association prediction	Singh and Lio, 2019		√	√
		Wang et al., 2019a		√	
		Han et al., 2019			√
	Cancer genetic judgment	Iglehart and Silver, 2009	√		
	Synthetic lethality prediction	Cai et al., 2020		√	
	Breast cancer metastasis prediction	Chereda et al., 2021			√
	Breast cancer subtype classification	Rhee et al., 2018		√	
	Disease protein judgment	Eyuboglu and Freeman, 2004	√		
	Discussion on the relationship between drug and disease	Bajaj et al., 2017		√	
	RNA classification	Rossi et al., 2019			√
	Predict the binding site of RNA protein	Uhl et al., 2019			√
		Yan et al., 2020			√
	RNA interaction prediction	Huang et al., 2019			√
	Targeted gene prediction of lncRNA	Zhao et al., 2020			√
	Biomedical data classification	Wang T. et al., 2020			√
Drug development and discovery	Protein structure prediction	Zamora-Resendiz and Crivelli, 2019	√		
	Protein design	Ingraham et al., 2019; Strokach et al., 2020			√
	Protein function prediction	Gligorijevic et al., 2019			√
		Fout et al., 2017		√	
	Protein functional sites prediction	Toomer, 2020	√		
	Interface prediction between protein pairs	Yao et al., 2020			√
	Protein–protein interaction network denoising	Liu X. et al., 2020		√	
	Prediction of the influence of protein mutation on binding	Cao and Shen, 2020	√		
	Protein docking model evaluation	Johansson-Åkhe et al., 2020			√
	Assessment of docked peptide conformations	Feng et al., 2018			√
	Drug–target interaction prediction	Gao et al., 2018; Miyazaki et al., 2020; Nguyen et al., 2021		√	
		Neil et al., 2018; Torng and Altman, 2019; Jiang et al., 2020; Zhong et al., 2020; Zhao et al., 2021			√
	Molecular structure coding	Duvenaud et al., 2015; Kearnes et al., 2016			√
	Drug properties prediction	Liu et al., 2019			√
	Esp surface detection of ligands	Rathi et al., 2019	√		
	Compound–protein interaction prediction	Li S. et al., 2020		√	
		Tsubaki et al., 2018			√
	Compound stability prediction	Li et al., 2019b			√
	*De novo* molecule design	Dai et al., 2018; Li et al., 2018a,b; You et al., 2018; Khemchandani et al., 2020			√
	Prediction of the association between miRNA and drug resistance	Huang et al., 2020		√	
	Prediction of the effect of drugs on cancer cell growth	Liu Q. et al., 2020; Singha et al., 2020			√
	Prediction of drug-induced liver damage	Hwang et al., 2020			√
	Side effects prediction between drugs	Zitnik et al., 2018		√	
	Drug similarity prediction	Ma et al., 2018		√	
	Drug combination design	Long et al., 2020		√	
	Drug recommendation	Mao et al., 2019		√	
	Alkaloid classification	Eguchi et al., 2019			√
	Product prediction of organic reactions	Coley et al., 2019			√
	Chemical network prediction	Li Q. et al., 2018		√	
Medical image processing	Image segmentation	Gopinath et al., 2019; Wang et al., 2019b; Tian et al., 2020a,b	√		
	Image classification	Shi et al., 2019; Zhou et al., 2019; Adnan et al., 2020	√		
	abnormal detection	Wu et al., 2019	√		
	Image visualization	Levy et al., 2020; Sureka et al., 2020			√
	Image enhancement	Hu et al., 2020	√		
	Image registration	Hansen et al., 2019	√		
	Image retrieval	Zhai et al., 2019	√		
	Surgical image analysis	Zhang et al., 2018	√		
	Disease prediction	Parisot et al., 2017; Kazi et al., 2018, 2019a,b, 2019c; Anirudh and Thiagarajan, 2019; Yang et al., 2019; Arya et al., 2020; Stankevičiūtė et al., 2020		√	
	Brain connection research	Ktena et al., 2017, 2018; Li X. et al., 2019a; Mirakhorli and Mirakhorli, 2019; Grigis et al., 2020; Zhang and Pierre, 2020; Zhang et al., 2021		√	
		Li et al., 2020		√	√

## Discussion and Future Research Directions

### The Problem and Trend of Methodology

Current GNNs have room for improvement in the methods of processing biological tasks. This section proposes the methodological problems and future development directions of GNNs for the three application fields of disease prediction, drug discovery, and biomedical imaging.

For disease prediction, improvements need to be made in three areas: the similarity evaluation of new nodes, the introduction of node attribute information, and heterogeneous information processing. First, most of the research in disease prediction adopted broad similarity methods. GNN models are used to extract the in-depth information of a heterogeneous network composed of disease semantic similarity, RNA functional similarity, and multiple association data. However, the construction of various similarity networks would have increased the complexity of the GNN model to a certain extent, and an efficient similarity evaluation paradigm needs to be improved for new diseases or RNA. In addition, more attention should be paid to the introduction of node attribute information into the modeling process, such as disease semantic features and RNA structural features, which can avoid the excessive dependence on associated information. Finally, for heterogeneous networks containing multi-source information, GNNs can deeply integrate their topological information. Nevertheless, current GNNs mainly focus on the processing of isomorphic graphs and cannot sufficiently capture the heterogeneity of nodes and edges in heterogeneous networks (Zhang C. et al., 2019). So, a new architecture needs to be studied, which can consider the feature of data in heterogeneous biological networks.

In drug discovery, the construction mode of chemical networks and the definition of molecular model structure need to be further explored. In the study of compound interactions, compounds and chemical networks are usually modeled as graphs. These graph-based methods have been successfully applied to the related tasks of chemical networks, but there are few studies that can simultaneously consider these two different types of graphs in an end-to-end manner. Harada et al. (2020) used molecular graphs as nodes in chemical networks and performed internal and outer convolution operations on them. The dual graph convolutional network can capture the feature of the individual molecular graph structure and the molecular relationship network simultaneously, making excellent results in dense networks. In addition, current molecular modeling is based heavily on the 2D graph structure, and the 3D structure that may affect the properties of molecules has rarely been considered. Therefore, the research of GNNs on the molecular 3D structure may be a future direction that has been neglected previously.

#### Multimodal Fusion

Graph neural networks also have limitations in multimodal data processing in medical imaging. The natural combination of multimodal deep learning (Ngiam et al., 2011) and multi-source omics data accelerates the development of bioinformatics. Some studies of GNNs in the multimodal fusion have been discussed in Section 4.3.3. It is not uncommon to find that the data is relatively balanced in these studies, but the involvement of unbalanced data cannot be avoided in actual tasks. Therefore, the method of processing unbalanced data in GNNs needs further research.

### The Problem and Trend Caused by Biological Data

Existing research on biomolecular networks has proved that many biomolecular networks have the properties of sparseness and scale-free nature. Sparseness is expressed as if the network size is *N*, then the number of edges is O(*N*) instead of *N*^2^, which results from a particular optimization in the long-term evolution of organisms. The scale-free nature is reflected in the degree distribution of these networks that obeys the power-law distribution, where most nodes have a small number of connections and a few nodes have a large number of connections. This characteristic demonstrates that a few nodes represented as biomolecules play a key role in the dynamic changes of biomolecular networks. For the problems of sparseness and scale-free nature of biomolecular networks, two methods—dropout and regularization—can be adopted to alleviate the overfitting caused by them. With a fixed probability, the dropout method randomly sets each dimension of the weight to zero during the training process so that the model only updates part of the parameters each time. As an example, the GCN model proposed by Cai et al. (2020) is based on the methods of fine-grained edge dropout and coarse-grained node dropout to making GCN learn a more stable representation in the process of continuous adaptation. Dropout can alleviate the instability when there is a fantastic amount of data in the training set; consequently, the dropout method is better suited to large data sets. The regularization method adds a regularization term to the loss function to limit the scale of parameters. In the study of disease–gene association prediction by Han et al. (2019), there were 3,209 diseases and more than 10,000 genes in the data set. Nevertheless, only 3,954 known disease–gene associations and a margin control loss function were defined to reduce sparse impact.

In addition, the collection of negative sample data is often ignored in the most current research, which leads to the fact that the biological data only contains positive samples. The lack of negative samples increases the difficulty of model training. Therefore, in the study of Eyuboglu and Freeman (2004), a simple method was proposed to solve the lack of negative labels, which randomly select *k* from the set of unlabeled nodes to mark them as negative. This seemingly unreasonable method causes most tags to be randomly selected, but in fact, randomly selected proteins may have a low correlation with disease. Hence, a certain number of random negative labels can be considered reasonable.

Finally, there is a large amount of noise information in the biomolecular network, so noise reduction processing is a very positive step for improving the model’s performance. For example, GAT can assign low weight to noisy data or directly eliminate the associations with correlation degrees lower than the threshold in the network. More methods for reducing noise are worth further development.

### The Lack of Interpretability

In bioinformatics, simply providing the computing results is just not far enough. The lack of interpretation is a persistent problem of the black box model like deep learning. As the entities and relationships in GNNs usually correspond to various types of objects that exist in the real world, then GNNs have abilities to support more interpretable analysis and visualization (Selsam et al., 2018). Take learning molecular fingerprint (Duvenaud et al., 2015) as an example; the fingerprint encoding method using neural graphs can take into account the similarity between molecular fragments to achieve a more meaningful feature representation, which is also ignored in traditional fingerprint encoding. In the prediction of metastasis for breast cancer patients (Chereda et al., 2021), the graph layer-wise relevance propagation was proposed to explain how GCN generates predictions based on patient-specific PPI sub-network data which could be potentially highly useful for the development of personalized medicine. In histological image analysis, Sureka et al. (2020) modeled histological tissue as a nuclear graph and established a graph convolutional network framework based on attention mechanism and node occlusion for disease diagnosis. This method visualized the relative contribution of each cell nucleus by a whole-slide image. In data analysis, GNNs generate relevant information for each data node, which makes the model more interpretative to some extent. Overall, the further exploration of interpretability in modeling biological networks by GNNs is still an essential direction of future research.

### The Exploration of Deep Structure

The deep network structure is more common in deep learning. For instance, a residual network (ResNet) that excels in image classification has 152 layers (He et al., 2016), but the layers of most networks are below three in the field of GNNs (Zhou et al., 2020). Experiments have shown that, as the number of network layers increases, the characteristics of all nodes will approach the same value, which will reduce network performance (Li Q. et al., 2018). However, deeper networks can provide larger parameter space and stronger representation capabilities, so the feasibility of a deep graph neural network deserves to be explored.

## Conclusion

Graph neural networks, as a branch of deep learning in non-Euclidean space, perform particularly well in various tasks that process graph structure data. In this paper, a systematic survey of GNNs and their advances in bioinformatics is presented from multiple perspectives. Three representative tasks are especially proposed based on the three levels of structural information that can be learned by GNNs: node classification, link prediction, and graph generation. Meanwhile, according to the specific applications for various omics data, we categorize and discuss the related studies in three aspects: disease prediction, drug discovery, and biomedical imaging. Finally, the limitations and future possibilities of applying GNNs to bioinformatics studies are illustrated.

Although GNN has achieved excellent results in many biological tasks at present, it still faces challenges in terms of low-quality data processing, methodology, and interpretability and has a long road ahead. We believe that GNNs are potentially a wonderful method that solves various biological problems in bioinformatics research. Furthermore, this paper can provide a valuable reference for new researchers joining the studies in this area.

## Data Availability Statement

The original contributions presented in the study are included in the article/supplementary material, further inquiries can be directed to the corresponding author/s.

## Author Contributions

LLia and X-MZ contributed to conceptualization, methodology, and writing – original draft preparation. LLiu and M-JT contributed to formal analysis and writing – review and editing. X-MZ contributed to the investigation and data curation. All authors have read and agreed to the published version of the manuscript.

## Conflict of Interest

The authors declare that the research was conducted in the absence of any commercial or financial relationships that could be construed as a potential conflict of interest.

## Publisher’s Note

All claims expressed in this article are solely those of the authors and do not necessarily represent those of their affiliated organizations, or those of the publisher, the editors and the reviewers. Any product that may be evaluated in this article, or claim that may be made by its manufacturer, is not guaranteed or endorsed by the publisher.
